# Injectable FHE+BP composites hydrogel with enhanced regenerative capacity of tendon-bone interface for anterior cruciate ligament reconstruction

**DOI:** 10.3389/fbioe.2023.1117090

**Published:** 2023-02-23

**Authors:** Eunshinae Cho, Yi Qiao, Changan Chen, Junjie Xu, Jiangyu Cai, Yamin Li, Jinzhong Zhao

**Affiliations:** Department of Sports Medicine, Shanghai Sixth People’s Hospital Affiliated to Shanghai Jiao Tong University School Of Medicine, Shanghai, China

**Keywords:** black phosphorous, anterior cruciate ligament reconstruction, tendon graft to bone tunnel healing, injectable hydrogel, FHE

## Abstract

Features of black phosphorous (BP) nano sheets such as enhancing mineralization and reducing cytotoxicity in bone regeneration field have been reported. Thermo-responsive FHE hydrogel (mainly composed of oxidized hyaluronic acid (OHA), poly-ε-L-lysine (ε-EPL) and F127) also showed a desired outcome in skin regeneration due to its stability and antibacterial benefits. This study investigated the application of BP-FHE hydrogel in anterior cruciate ligament reconstruction (ACLR) both in *in vitro* and *in vivo*, and addressed its effects on tendon and bone healing. This BP-FHE hydrogel is expected to bring the benefits of both components (thermo-sensitivity, induced osteogenesis and easy delivery) to optimize the clinical application of ACLR and enhance the recovery. Our *in vitro* results confirmed the potential role of BP-FHE *via* significantly increased rBMSC attachment, proliferation and osteogenic differentiation with ARS and PCR analysis. Moreover, *In vivo* results indicated that BP-FHE hydrogels can successfully optimize the recovery of ACLR through enhancing osteogenesis and improving the integration of tendon and bone interface. Further results of Biomechanical testing and Micro-CT analysis [bone tunnel area (mm2) and bone volume/total volume (%)] demonstrated that BP can indeed accelerate bone ingrowth. Additionally, histological staining (H&E, Masson and Safranin O/fast green) and immunohistochemical analysis (COL I, COL III and BMP-2) strongly supported the ability of BP to promote tendon-bone healing after ACLR in murine animal models.

## 1 Introduction

Anterior cruciate ligament (ACL) is an essential ligament responsible for the maintenance and stability of the knee joint (Duthon et al., 2006; Zantop et al., 2006). However, this ligament is associated with limited healing ability due to the lack of proper vascularization. Thus, ACL reconstruction (ACLR) is the recommended surgical approach in the case of injury (Yao et al., 2021).

Generally, the human bone structure is unique and contains a variety of molecules and cells such as type I collagen, calcium, phosphate, osteoblasts, osteocytes and osteoclasts (Rho et al., 1998; Mohammadi et al., 2018). Such components play an important role in the formation and regeneration of bones, guaranteeing proper functioning and regulation (Qing et al., 2020). Therefore, research focuses on investigating the bone structure and optimal strategies to enhance its recovery (Lopes et al., 2018). Points of interest include the value of certain materials in stimulating recovery and the optimal delivery methods to maximize effects and shorten the time of recovery (Williams, 2008; Mondschein et al., 2017; Wang et al., 2022).

Considering the importance of phosphorus in the human body (making up to 1% of total body mass) (Yang et al., 2018a; Ouyang et al., 2020), especially in human bones and teeth (Cui et al., 2016; González Díaz et al., 2018), many studies focused on this element and its role in bone recovery and generation. Of the different types of phosphorous, black phosphorous (BP) has been presented as a promising element with great potential. Synthesized from white phosphorous, BP proved to have better stability and compatibility in bioengineering applications and is suggested as an important osteogenic inducer with great potential in the treatment of bone injury due to its features of single element structure and easy degradability (Doganov et al., 2015; Huang et al., 2019; Cheng et al., 2020; Pandey et al., 2020).

The further invention of BP nanosheets brought additional features such as optimized electron mobility, charge carrier capability, optical properties, enhanced mineralization and reduced cytotoxicity which led to great enhancements in the field of bone regeneration (Dadsetan et al., 2012; Li et al., 2014; Erande et al., 2016; Zhu et al., 2016; Yang et al., 2018a; Yang et al., 2018b; Wang et al., 2018; Qing et al., 2020; Sun et al., 2020).

The main mechanism of BP nanosheets is related to its *in vivo* degradation that enhances mineralization and leads to better adhesion and differentiation of bone cells. Thus, it translates into accelerated proliferation and tissue regeneration (Goretti Penido and Alon, 2012; Rashdan et al., 2016; Qing et al., 2020). That led to the idea of BP nanosheets incorporation in novel delivery systems such as bio-degradable hydrogels to optimize osteogenesis regardless to the presence of inductive elements (Qing et al., 2020). Such incorporation can also present an efficient sustained *in vivo* release of BP to increase efficacy and limit general side-effects (Cheng et al., 2020).

The benefits of hydrogel application (such as the easy injectability, biocompatibility, degradability and limited toxicity) (Rowley et al., 1999; Hennink and van Nostrum, 2002; Hoffman, 2002; Zhang, 2003; Hou et al., 2019) favored it as a delivery vessel in relevant researches. Relevant investigations include the use of hydrogel in ACLR animal models (Chen et al., 2008) and the combination of gelatin methacryloyl (GelMa) with BP nanosheets (Huang et al., 2019; Miao et al., 2019).

Among the different types of hydrogels, those responsive to external stimulation (light, temperature, pH, *etc.*) grabbed the attention (Klouda and Mikos, 2008). Of which, thermo-responsive hydrogels have been strongly present in therapeutic applications such as drug and cell delivery (Stile et al., 1999; Guan et al., 2008; Vermonden et al., 2008; Fundueanu et al., 2009; Misra et al., 2009), tissue engineering (Tang et al., 2010) and myocardial injections (Fujimoto et al., 2009) due to their minimal invasiveness and toxicity, convenient preparation, and long-term effectiveness (Lin et al., 2014).

FHE hydrogel (F127/OHA-EPL) is a thermo-responsive hydrogel (mainly composed of oxidized hyaluronic acid (OHA), poly-ε-L-lysine (ε-EPL) and F127) which showed a desired outcome in skin regeneration due to its stability and antibacterial benefits. In addition to thermal sensitivity, FHE hydrogel provides advanced biocompatibility and cellular adhesion (Wang et al., 2019).

In light of the great potential of BP and FHE hydrogel application, and since neither has been explored thus far in ACLR research, this study investigated the application of BP-FHE hydrogel after ACLR to address their effects on tendon and bone healing. This combination is expected to bring the benefits of both components (thermo-sensitivity, induced osteogenesis and easy delivery) to optimize the clinical application of ACLR and enhance recovery.

## 2 Methods

### 2.1 Hydrogel preparation and characterization

#### 2.1.1 Materials

BP nanoplates were obtained from HWRK Chem (Beijing, China). Sodium hyaluronate (HA, Mw = 1.5×10^6^) was purchased from Shanghai Yuanye Biotechnology Co. (Shanghai, China). Sodium periodate (NaIO_4_), F127, and ε-polylysine (EPL) were gained from Aladdin Reagent Co. (Shanghai, China). All materials and solvents were used as received without any further purification unless otherwise noted.

#### 2.1.2 Synthesis of oxidized hyaluronic acid (OHA)

Hyaluronic acid (HA) was oxidized by sodium periodate (NaIO_4_) to obtain oxidized hyaluronic acid (OHA). The solution of 1% (w/v) was prepared by dissolving 2 g sodium hyaluronate in 200 mL deionized water at room temperature for 24 h. Then, 1.08 g sodium periodate was weighed and completely dissolved in 10 mL deionized water, and slowly added into sodium hyaluronate solution, the whole process was operated away from light. The mixed solution was stirred at room temperature away from light for 2 h, and then 2 mL glycol was added to stop the reaction for 1 h. The final product solution was purified by dialysis in deionized water for 72 h. Finally, the purified solution was pre-frozen in a −80°C refrigerator, and the dried OHA was purified by freeze-drying machine under vacuum drying conditions.

In order for oxidative degree to accurately present dialdehyde content, we adopted the definition of the oxidized uronic acid unit to total hyaluronic acid unit mole ratio. Iodometric titration and hydroxylamine hydrochloride were used (according to previous method) to measure OHA’s oxidative degree (Balakrishnan and Jayakrishnan, 2005; Yuan et al., 2017). Oxidative degree’s maximum was 75.8% when the mole ratio (NalO4/hyaluronic acid repeat unit) was 1.5.

#### 2.1.3 Preparation of injectable FHE + BP composites hydrogel

OHA was freeze-dried and dissolved into an 80 mg/mL solution using distilled water as solvent. ε-EPL was dissolved into a solution by using the same method with a concentration of 50 mg/mL and 100 mg/mL. Then F127 was dissolved into a 400 mg/mL solution under 4°C according to the volume ratio of F127: ε-EPL: OHA as 3:1:1. The F127 solution and the ε-EPL solution were sequentially mixed at 4°C, and the OHA solution was added after mixing evenly, the solution was then put in a thermostatic shaker for gelation, hydrogels were named as FHE.

BP nanoplates combined with FHE hydrogel were prepared similarly to the above procedure of FHE. After mixing F127 and ε-EPL solutions at 4°C, BP was dispersed in the mixed solution with a relative mass fraction [W_BP_ (W_BP_ + W_FHE_)] of 5%. After mixing, the solution was continuously stirred using a stirrer for 2 days at 37°C until completely dissolved to obtain FHE + BP composites hydrogel.

#### 2.1.4 Characterization and testing of hydrogel

Attenuated total reflectance-Fourier transform infrared spectrometer (ATR-FITR, Thermo Nicolet, United States) was employed to characterize the chemical structure of synthetic gels in the range of 400–4,000 cm^-1^ under a resolution of 4 cm^-1^. Synthetic gels were characterized by ^1^H spectrum nuclear magnetic resonance (^1^H-NMR, AVANCE-400MHz, Bruker, Switzerland) with DMSO-d_6_ as solvent. The morphology and surface structure of gels were carried out using a scanning electron microscope (SEM, Phenom XL, Netherlands) operating with sputter gold plating for 35 s at 5 mA at an accelerating voltage of 10 kV. ImageJ (National Institutes of Health, United States) was adopted to determine the pore diameter of gels.

#### 2.1.5 Swelling ratio and water retention ratio testing of hydrogel

Phosphate buffer solution (PBS) was dropped into the test tube with hydrogels for several times until the volume no longer changed and then the mass was weighed. The hydrogel was lyophilized and weighed, and its water absorption ratio was computed through Eq. 1:

$$Swelling\;ratio=\left(W_{t}-W_{0}\right)/W_{0}\times 100\%$$ (1)

When the hydrogel was at swelling equilibrium, the weight was marked as W_t_, and when it was lyophilized, the weight was marked as W_0_.

The prepared hydrogel was weighed, then placed in an environment of 37°C with a relative humidity of 70%. After 12 h or 24 h of air-dry operation, the weight of hydrogel was recorded, respectively. Water retention ratio was defined as Eq. 2:

$$Water\;retention\;ratio=\left(W_{t}-W\right)/\left(W_{0}-W\right)\times 100\%$$ (2)

At the beginning, the initial weight of the hydrogel was recorded as W_0_; Wt was the weight of the hydrogel after 12 h or 24 h of air-dry operation; The weight of hydrogel after lyophilized was recorded as W.

### 2.2 *In vitro* experiments

#### 2.2.1 Rat BMSCs (rBMSCs) isolation and culture

First, the bone marrow–derived mesenchymal stem cells (BMSCs) were isolated from Sprague Dawley (SD) rats based on a previously described protocol (Li et al., 2013). Considering mesenchymal stem cells ability of differentiation, their application in regeneration medicine has been increasingly growing. Many studies counted on MSCs (from different origins) to successfully investigate and promote tendon-bone healing (Chen et al., 2021). Obtained cells were seeded on culture plates and cultured in a complete medium containing α-MEM, 10% FBS, and 1% penicillin/streptomycin (all from Gibco, United States). Plates were incubated with 5% CO_2_ at 37°C and culturing medium was changed once every 2 days. Propagation into new plates was carried at 80% confluence and further experiments were only carried after three propagations. Approval by Shanghai Sixth People’s Hospital Affiliated to Shanghai Jiao Tong University School of Medicine Ethics Committee (No. DWSY 2021-0127) was obtained for all carried animal experiments.

#### 2.2.2 Proliferation and attachment of rBMSCs

Cytotoxicity of BP nanosheets and FHE hydrogel on BMSCs was investigated by cell counting kit-8 (CCK-8, Beyotime, China). First, BP solution (0.125 mg/mL) was added to hydrogel solution (1:10) then vacuum dried. Then, 1 mg of vacuum dried powder was added to 1 mL of medium. After that, a leaching solution was prepared by adding 10 μL of the previous solution to 1 mL of medium. An additional leaching solution was prepared using vacuum dried hydrogel powder (10 μL/mL). Leaching solutions were stored at 4°C after being sterilized and filtered.

A 96-well plate was used to seed BMSCs (1 × 10^3^ cells per well). When the cells were attached, the different leaching solutions were used to replace the original medium and then refreshed every other day. For the control group, the medium was used alone (no leaching solution was added). Incubated for 1, 3 and 5 days, cells were then washed (PBS/2 times) and cultured in medium and CCK-8 solution (10:1 (v/v)) (2h, 37°C, 5% CO_2_). A microplate reader (Thermo Scientific, United States) was used to determine the optical density (OD) (450 nm absorbance).

As for cell viability analysis, rBMSC seeding in 24-well plate was carried and Propidium iodide (PI) (1 μl/mL) and AM (1 μl/mL) (Live/Dead viability/Cytotoxicity Assay Kit/Beyotime, China) were mixed in PBS and used to incubate (30 min) and stain live/dead cells. After the incubation, Zeiss 880 fluorescence microscope (Zeiss, Germany) was used to capture fluorescent images.

#### 2.2.3 Alizarin red S (ARS) staining

First, cells were washed and fixed in PBS buffer for 4 h (4°C, 2.5% glutaraldehyde) before being washed again. Different solutions were added based on the group and refreshed regularly with Osteogenesis induction medium (OIM) (10% FBS +0.1 μM dexamethasone +50 μg/mL ascorbic acid +10 mM sodium β-glycerophosphate +1% penicillin/streptomycin + DMEM). After 14 days, ARS (Sigma-Aldrich, Germany) staining for 30 min in room temperature (20 mg/mL ARS, pH 4.2) was used to evaluate bone-like inorganic calcium deposits. Cells were then washed until all ARS dye was removed from washed liquid and quantitative analysis was carried. Using 10% (w/v) cetylpyridinium chloride (Sigma Aldrich, United States), optical density at 562 mm was measured and ImageJ software was used to complete the analysis.

#### 2.2.4 Real-time quantitative polymerase chain reaction (RT-qPCR)

mRNA expression of COL I, RUNX-2, and OCN (osteogenic-relevant genes) was detected through RT-qPCR. First, 6-well plate was used to seed rBMSCs for 14 days. OIM was added and refreshed every other day. Then, Trizol-up (EZBioscience) was used to extract total RNA and 4X Reverse Transcription Master Mix (EZBioscience) to convert it to complementary DNA. Applied Biosystems 7500 Real-Time PCR system (2 X SYBR Green qPCR Master Mix (EZBioscience)) was used to perform RT-PCR following the protocol of manufacturer. Expression levels’ normalization was based on β-actin expression and 2^−ΔΔCT^ was used to calculate expression values. Table 1 describes the primers for RT-qPCR.

**TABLE 1:** Gene-specific primers for COL1, RUNX-2, OCN and β-actin.

Gene	Primer
COL1	5′- CTG​GGT​GGG​AGA​GAC​TGT​T-3′ (forward)
	5′- CGG​TGA​CAC​ACA​AAG​ACA​AG-3′ (reverse)
RUNX-2	5′- ATC​ATT​CAG​TGA​CAC​CAC​CAG-3′ (forward)
	5′-GTA​GGG​GCT​AAA​GGC​AAA​AG-3′ (reverse)
OCN	5′-CCT​CTC​TCT​GCT​CAC​TCT​GCT-3′ (forward)
	5′-CTT​ACT​GCC​CTC​CTG​CTT​G-3′ (reverse)
β-actin	5′-CCT​CTA​TGC​CAA​CAC​AGT-3′ (forward)
	5′-AGC​CAC​CAA​TCC​ACA​CAG-3′ (reverse)

### 2.3 *In vivo* experiments

This study included 72 SD rats (males, 12-13 weeks, 280–320 g) and divided them into control group (*n* = 24), FHE group (*n* = 24) and FHE + BP group (*n* = 24) (Oka et al., 2013). Unilateral ACLR was carried out in all rats by two investigators and each group was further divided into 4-week and 8-week subgroups. Euthanasia was carried out after 4 or 8 weeks (based on the group) *via* a CO_2_ overdose and biomechanical testing, micro-CT and histological analysis were carried out to assess collected samples of the femur-graft-tibia complex.

#### 2.3.1 Surgical procedure

Intraoperative 3% pentobarbital injection (1.0 mL/kg) was first used for anesthesia *via* intraperitoneal injection and the skin of lower limbs was shaved and sterilized. Then, a harvest of ipsilateral flexor digitorum longus tendons was completed (from the lateral aspect of ankle joints) and muscles on harvested grafts were removed. The knee was exposed through medial parapatellar arthrotomy and a lateral dislocation of the patella exposed native ACL. A careful excision of the native ACL was completed and confirmed after the tibia was translated anteriorly. After the knee was flexed (90°), tibial and femoral tunnels (diameter: 1.5 mm, length:7 mm) were created by 1.5 mm diameter Kirschner wire starting from the original ACL footprint to the tibia’s medial side (tibial tunnel) or the femoral condyle’s anterolateral side (femoral tunnel) (Lui et al., 2014). A 4-0 Ethibond (Ethicon) was used to attach one side of the graft and drag it into the tunnel. Previously stored at 4° FHE (or FHE + BP) were injected into tunnels before grafts’ immediate placement (dragging). The knee joint was flexed to 30° and 4N graft pretention was applied before the suturing of grafts to the surrounding periosteum at both tibial and femoral ends was completed. Layered wound closure was then carried and the anterior stability of the knee was validated by Lachman test. Intramuscular anti-infectious injections (penicillin, 50 KU/kg) were given to animals before returning to cages and being allowed free movement.

#### 2.3.2 Macroscopic observation

At two time points (4 and 8 weeks), an assessment of infection in the wound site was carried. Afterwards, observed knee joints were fully exposed and an assessment of graft was completed by two independent investigators. Scoring criteria included the stiffness and integration of the graft with surrounding tissues in addition to the appearance and color of articular surface. Details of the scoring system are provided in Supplementary Table S1 (Cheng et al., 2016).

#### 2.3.3 Micro-CT scanning

At 4 or 8 weeks after the operation, animals were sacrificed to collect samples. Femur-tibia complex samples (*n* = 6/group) were thawed and all structures and soft tissues were removed, maintaining only the reconstructed ACL graft. Samples were then frozen (−80°C) until further use. After being brought to room temperature, samples were scanned perpendicularly to the long axis of bone tunnel (spatial resolution: 18 μm, Skyscan 1176 micro-CT imaging system (Bruker, Kontich, Belgium)). Bone ingrowth was analyzed through focusing on cylinder-shaped region of interest (ROI) (diameter: 2 mm, height: 3 mm) and mean cross-sectional areas (mm^2^) of bone tunnels and tunnels’ bone volume/total volume (BV/TV) ratios were calculated to complete the quantitative analysis (Sun et al., 2019; Xu et al., 2022a).

#### 2.3.4 Biomechanical testing

After micro-CT scanning, Femur-tibia complex samples were used to complete biomechanical testing. Material testing machine (Model 2712-004; Instron Corp) was used to test the healing interface. The tested joint was mounted and both bone tunnels were aligned with the tensile load direction (Lui et al., 2014; Xu et al., 2022a). After that, preconditioning (5 cycles (maximum displacement of 0.5 mm) was completed. The failure mode and ultimate failure load was investigated by applying loads from 0-N to 0.5-N with 3 mm/min displacement rate (until failure). Failure was represented by the presence of ruptured graft or pulling out of the tunnel (Cai et al., 2021a). Samples were moisturized (saline solution) during testing and stiffness was determined through load-displacement curve.

#### 2.3.5 Histological analysis and immunohistochemical staining

Samples were fixed (10% formalin, 36 h) then decalcified (10% EDTA, 6 weeks) before a dehydration and paraffin embedding were carried. Then, samples were sliced (5μm, SM2500; Leica, Nussloch, Germany) parallelly to tunnels’ longitudinal axis and fixed on glass slides (40°F oven). Standard Hematoxylin and eosin (H&E) staining was completed to evaluate graft-bone interface.

Patterns of intra-articular collagen alignment were visualized by Masson’s trichrome staining and Safranin O/fast green staining was also carried to observe fibro-cartilage formation patterns and glycosaminoglycans (GAGs) content (Chen et al., 2021).

All staining procedures were carried based on manufacturer’s instructions before an inverted light microscopy (Leica DM4000 B, Germany) was used for observation and Leica DFC420C camera (Leica Microsystems GmbH) to capture images.

Obtained results were analyzed and quantified by two observers. Three parameters (fibrocartilage formation, new bone formation and graft bonding to adjacent tissues) were considered in the final scoring (0-3 points/item, 0-9 points for total score) with higher scores representing enhanced results. Details of the scoring system are provided in Supplementary Table S2 (Cheng et al., 2016).

Immunohistochemical staining (IHC) for COL I, COL III and BMP-2 was carried. First, samples’ dewaxing and rehydration were carried before antigen-retrieval. Then, 0.3% hydrogen perioxide (20 min) and 2% bovine serum albumin (1 h) were used for blocking and primary anti-body incubation was carried over-night (4°C). Secondary antibody was used for incubation for 1 h at 37°C. Samples were then washed. Finally, observation of obtained images was completed under a light microscopy (Leica DM4000 B, Germany).

### 2.4 Statistical analysis

GraphPad Prism 9 (California), Origin 8.0 statistical software (Origin Lab Inc., United States), ANOVA and Tukey’s test were applied to statistically analyze data. All data are expressed as mean ± standard deviation (Mean ± SD). *p*-values < 0.05 (*) were deemed to be statistically significant.

## 3 Results

### 3.1 Physicochemical properties of hydrogels

**FIGURE 1:**
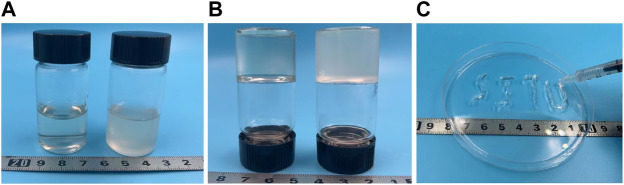
Optical picture of hydrogel **(A)** FHE hydrogel (left), FHE + BP hydrogel (right) **(B)** Gelation FHE hydrogel (left), FHE + BP hydrogel (right) **(C)** Injectable FHE hydrogel.

**FIGURE 2:**
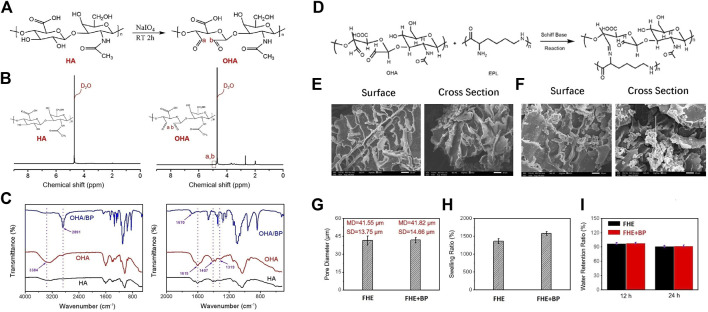
Physicochemical properties of hydrogels **(A)** The synthetic reaction mechanism route of OHA **(B)** 1H-NMR spectrum of HA and synthetic OHA **(C)** FTIR spectrum of HA, OHA, and OHA/BP **(D)** Schiff base reaction mechanism route between OHA and EPL **(E, F)** The surface and cross section SEM images of FHE hydrogel and FHE + BP hydrogel, respectively **(G)** The pore diameter of FHE hydrogel and FHE + BP hydrogel **(H)** The swelling ratio of FHE hydrogel and FHE + BP hydrogel **(I)** The water retention ratio of FHE hydrogel and FHE + BP hydrogel in an environment of 37°C with a relative humidity of 70% for 12 h and 24 h, respectively.

As mentioned earlier, NaIO_4_ was used to oxidize HA into OHA which contains side chain aldehyde group. Figure 1 shows the optical pictures of the different hydrogels. The reaction mechanism is shown in Figure 2A. Since the degree of oxidation of HA to OHA needs to be controlled artificially, the mole ratio of NaIO_4_ and HA was set as 1:1, and the oxidation time was 2 h.

After the oxidation, ^1^H-NMR spectrum test was applied to analyze the variation of HA absorption peak before and after modification (Figure 2B). Compared to the absorption peak on the HA ^1^H-NMR spectrum, the aldehyde group revealed a characteristic peak at the chemical shift *δ* = 5.0–5.1 ppm of OHA, indicating a successful synthesis of OHA.

After the successful synthesis, BP nanoplates were homogenized with OHA. The infrared spectrum test results revealed that compared to HA, OHA showed a new absorption peak at 1,729 cm^-1^, corresponding to the stretching vibration of the double bond of the aldehyde group -C=O (Figure 2C). The FTIR spectrum of OHA/BP also presented the existence of BP in OHA/BP composites hydrogels.

The synthesis mechanism of FHE hydrogel is shown in Figure 2D. As for the analysis of SEM images of FHE and FHE + BP hydrogels, our results showed that the pore size of the hydrogel with BP nanoflakes and the FHE hydrogel are not very different (Figure 2E–G), indicating that BP is evenly dispersed in the FHE hydrogel since the pore size of the hydrogel measured by the ImageJ software before and after the adulteration was basically unchanged. In addition, As shown in Figures 2H, I, the water absorption of the hydrogel increased significantly after the addition of BP.

### 3.2 *In vitro* experiments

#### 3.2.1 Proliferation and attachment of rBMSCs

**FIGURE 3:**
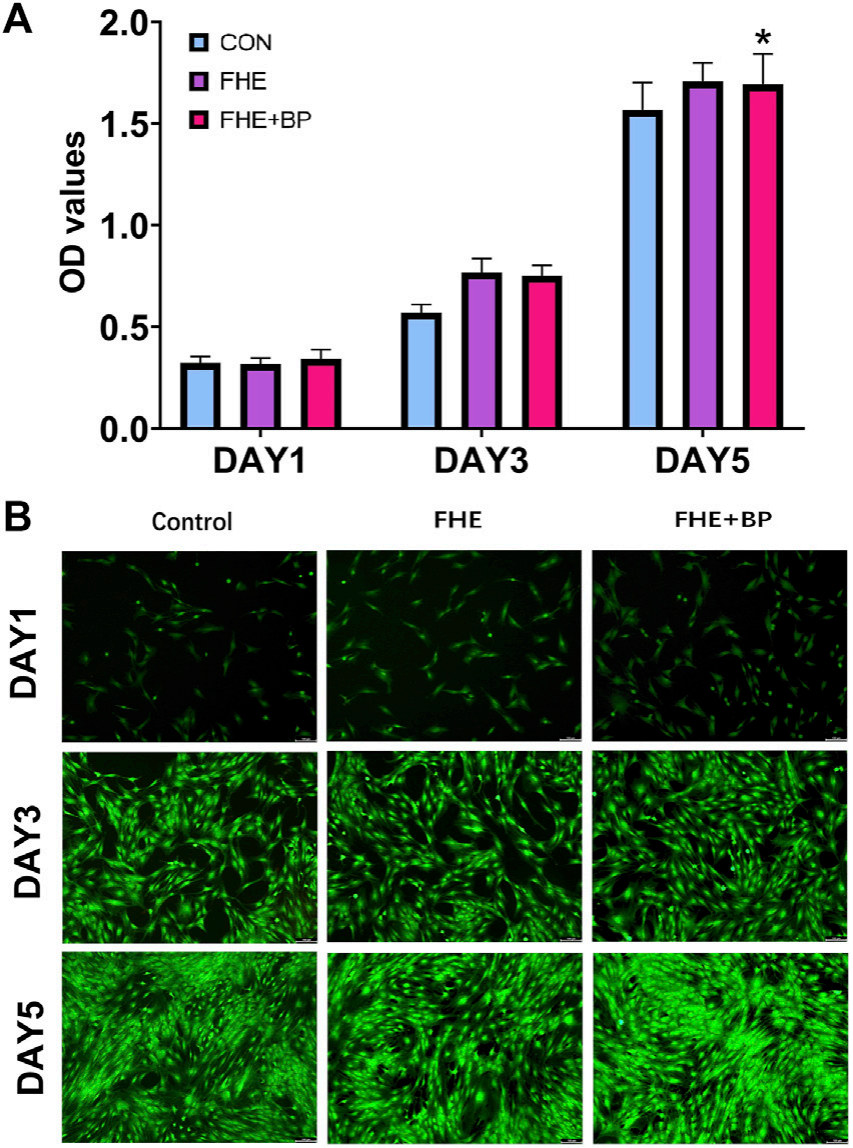
Evaluation of biocompatibility and cytotoxicity of BP and FHE **(A)** CCK-8 assay and observed OD values of control, FHE and FHE + BP groups at day 1, 3 and 5 **(B)** Fluorescent images of live/dead cellular staining at day 1, 3 and 5 in all three groups. *p < 0.05 compare to control group.

The rBMSCs were successfully cultured and propagated (X3) before carrying further experiments. We started our *in vitro* experiments by investigating the biocompatibility and cytotoxicity of BP and FHE hydrogel using CCK-8. Three groups were included in this investigation: control group, FHE and FHE + BP groups (10 μL/mL) and OD values on days 1, 3 and 5 were observed (Figure 3A). Our results showed no significant differences among groups at day 1; however, OD values differed significantly at day 3 and 5. As expected, in comparison with day 1, all groups had significantly higher OD values at day 3 (mean differences: CON: 0.2474, *p* < 0.0001; FHE: 0.4488, *p* < 0.0001; FHE + BP: 0.4087, *p* < 0.0001) and day 5 (mean differences: CON: 1.245, *p* < 0.0001; FHE: 1.39, *p* < 0.0001; FHE + BP: 1.352, *p* < 0.0001). On day 3, both FHE and FHE + BP groups showed significantly higher OD values than the control group (mean differences: FHE: 0.1968, *p* = 0.0001; FHE + BP: 0.1804, *p* = 0.0005). Similar results were observed at day 5 (mean differences: FHE: 0.1407, *p* = 0.0156; FHE + BP: 0.1261, *p* = 0.0452). No significant difference was observed between FHE and FHE + BP group at either day 3 or day 5. Our live/dead staining results (Figure 3B) further supported the findings, which showed a stronger proliferation of cells (green staining) at day 5 in FHE + BP group when compared to FHE and control groups.

#### 3.2.2 Osteogenic differentiation

**FIGURE 4:**
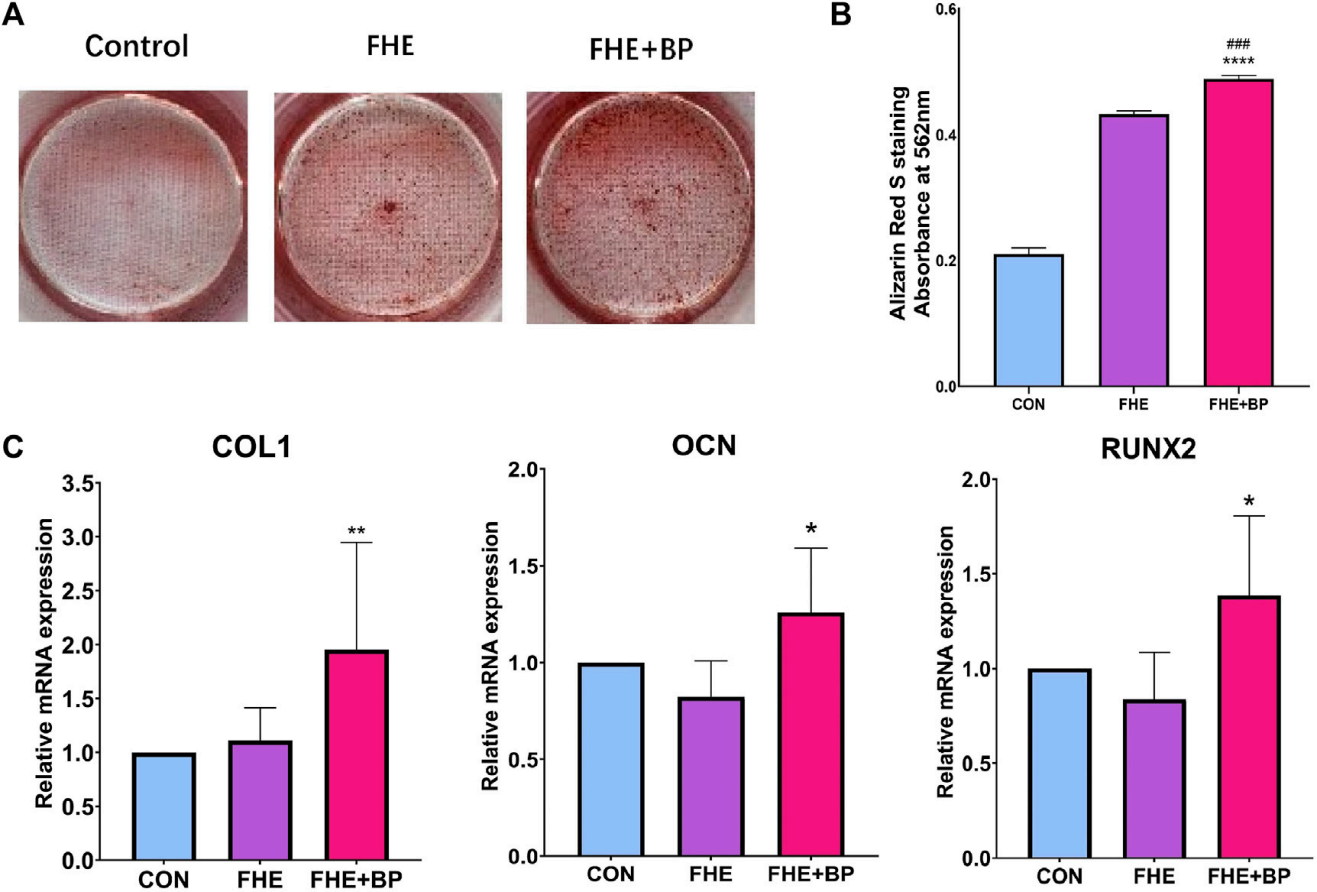
Analysis of osteogenic differentiation **(A)** Images of Alizarin Red S staining of all three groups after 14 days of cell culture **(B)** Absorbance and optical density at 562 mm in the three groups after ARS staining **(C)** mRNA expression levels of COL-1, OCN and RUNX2 in the three groups after 14 days of cell culture. *p < 0.05 compare to control group, **p < 0.01 compare to control group, ****p < 0.0001 compare to control group, ###*p* < 0.001 compare to FHE group.

To confirm osteogenesis differentiation, ARS staining was first used to evaluate bone-like inorganic calcium deposits. The general observation revealed a stronger ARS staining in FHE + BP group (represented by the red color) (Figure 4A). As shown in Figures 4A, B significant effect of BP on formation at day 14 was observed. The analysis of optical density at 562 mm showed that OD value of FHE + BP group was significantly higher than those of the other two groups with a mean difference of 0.056 with FHE group (*p* = 0.0002) and 0.2788 with control group (*p* < 0.0001).

The osteogenic effects of BP were further proved by RT-qPCR. Three osteogenic relevant genes were included (COL1, OCN and RUNX-2). The results showed a significant increase of expression in FHE + BP group of all three genes at day 14 compared to FHE and control groups (Figure 4C). For COL1, no significant difference was found between the control and FHE groups (*p* = 0.9165), while BP was significantly higher than both control group (mean difference: 0.954, *p* = 0.0068) and FHE group (mean difference: 0.8414, *p* = 0.0173). Similar results were observed for OCN (CON vs FHE: 0.1764, *p* = 0.2233; CON vs FHE + BP: −0.2601, *p* = 0.048; FHE vs FHE + BP: −0.4364, *p* = 0.0008) and RUNX-2 (CON vs FHE: 0.1618, *p* = 0.4549; CON vs FHE + BP: −0.3858, *p* = 0.0206; FHE vs FHE + BP: ‒0.5477, *p* = 0.0011).

### 3.3 *In vivo* experiments

#### 3.3.1 Macroscopic observation

**FIGURE 5:**
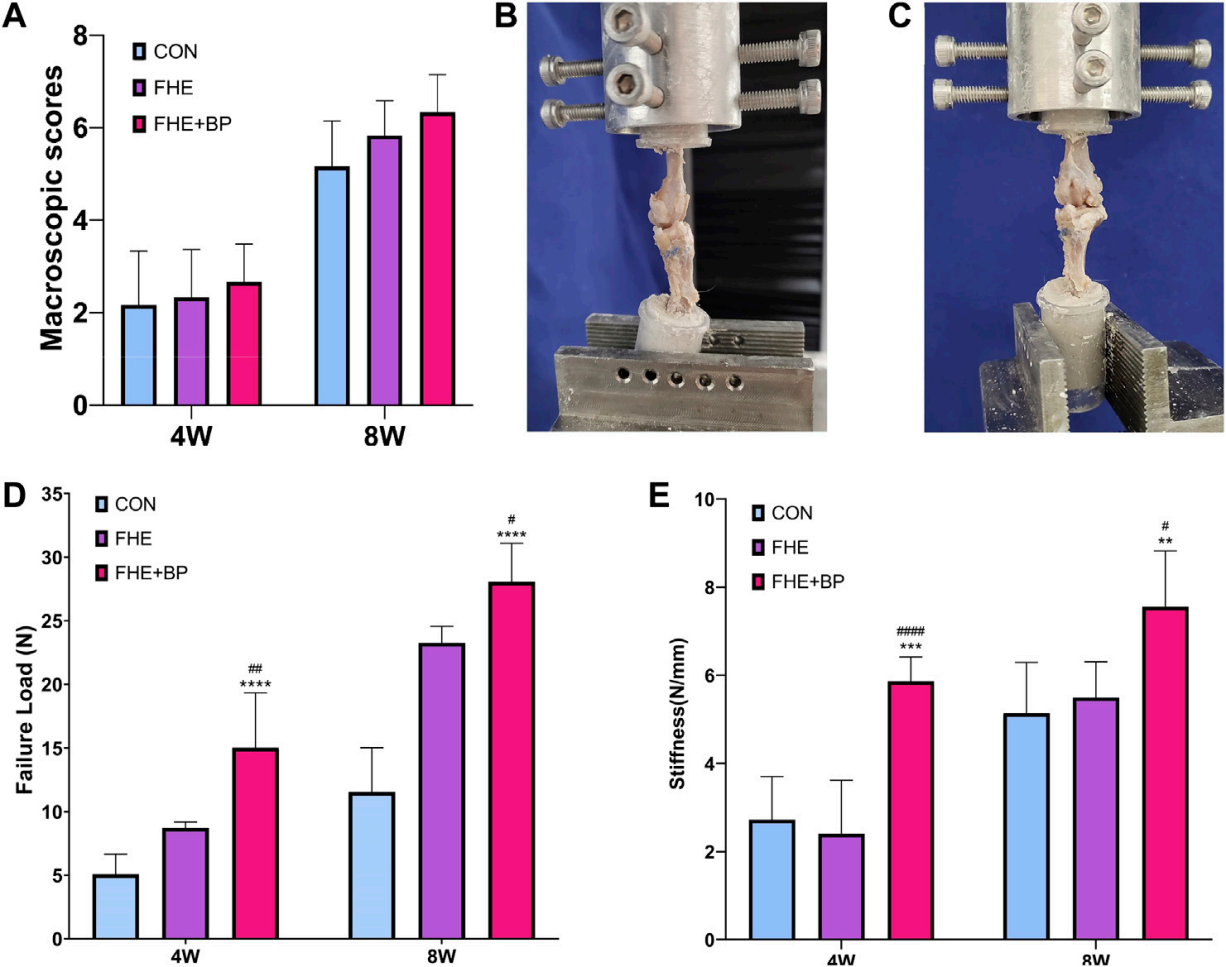
Macroscopic observation and biomechanical testing **(A)** Macroscopic scores of all three groups after 4 and 8 weeks **(B, C)** Representative images of the procedure of biomechanical testing **(D)** Comparison of failure load (N) in all three groups after 4 and 8 weeks **(E)** Comparison of stiffness (N/mm) in all three groups after 4 and 8 weeks **p < 0.01 compare to control group, ***p = 0.001 compare to control group, ****p < 0.0001 compare to control group, #*p* < 0.05 compare to FHE group, ##*p* < 0.005 compare to FHE group, ####*p* < 0.0001 compare to FHE group.

We started our *in vivo* investigation by completing a macroscopic observation of grafts at 4 and 8-week timepoints. No signs of infection were observed at any time point and all grafts appeared fully intact. As for detailed scores, although no significant differences were recorded among groups, higher scores were observed at 4 and 8 weeks in FHE + BP (compared to control and FHE groups) due to enhanced stiffness, integration, appearance and color of graft (4 weeks: CON = 2.167, FHE = 2.333, FHE + BP = 2.667; 8 weeks: CON = 5.167, FHE = 5.833, FHE + BP = 6.333) (Figure 5A).

#### 3.3.2 Biomechanical testing

Biomechanical testing of failure load and stiffness after 4 and 8 weeks was carried in all three groups (Figures 5B, C). At week 4, a significantly higher failure load was recorded in FHE + BP group (15.02 N) in comparison with the control group (5.07 N, *p* < 0.0001) and FHE group (8.723 N, *p* = 0.0045). Similar results were observed at week 8 (FHE + BP = 28.06 N; CON = 11.54, *p* < 0.0001; FHE = 23.25, *p* = 0.0474) (Figure 5D). Results of stiffness (N/mm) also favored FHE + BP group after 4 and 8 weeks (4 weeks: FHE + BP = 5.863; CON = 2.722, *p* = 0.0001; FHE = 2.404, *p* < 0.0001) (8 weeks: FHE + BP = 7.555; CON = 5.136, *p* = 0.0038; FHE = 5.494, *p* = 0.0179) (Figure 5E).

#### 3.3.3 Micro-CT scanning

**FIGURE 6:**
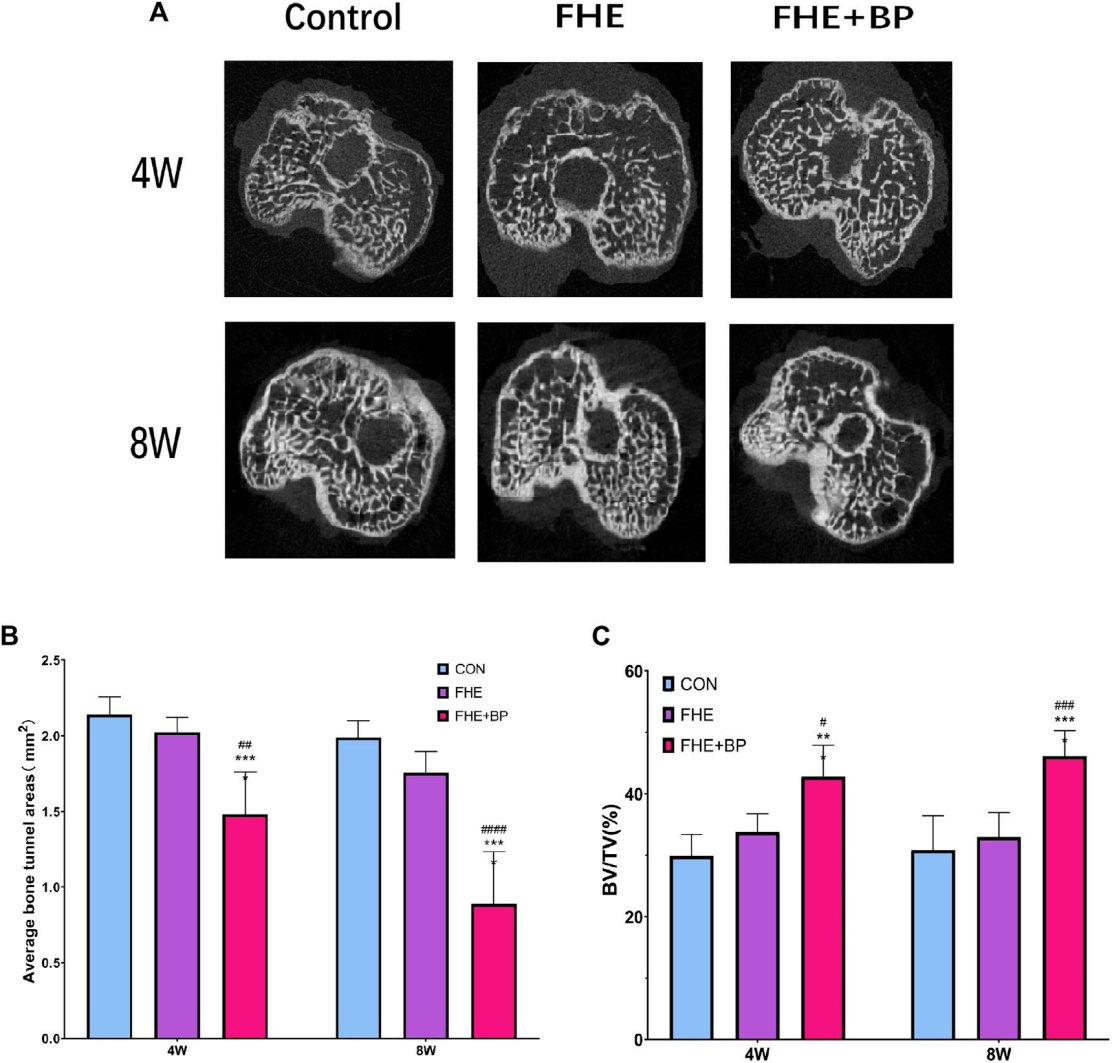
Micro-CT scanning **(A)** Representative images of cross-sectional areas of bone tunnels in all three groups after 4 and 8 weeks **(B)** Comparison of average bone tunnel areas (mm^2^) of the three groups at 4 and 8 weeks **(C)** Comparison of BV/TV (%) of the three groups at 4 and 8 weeks ***p = 0.002 compare to control group, ****p < 0.0001 compare to control group, #*p* = 0.0129 compare to FHE group, ##*p* = 0.001 compare to FHE group, ###*p* = 0.001 compare to FHE group, ####*p* < 0.0001 compare to FHE group.

Micro-CT scanning was completed to evaluate the formation of bone in the tunnels and two parameters (average bone tunnel area (mm^2^) and BV/TV (%)) were calculated at 4 and 8-week time-points (Figure 6A). Our results showed that after 4 weeks, the average bone tunnel area was 1.482 mm^2^ for FHE + BP group, compared to 2.14 mm^2^ for the control group (*p* < 0.0001) and 2.023 mm^2^ for FHE group (*p* = 0.001). After 8 weeks, the groups’ average bone tunnel areas were 0.8887 mm^2^ for FHE + BP group, 1.989 mm^2^ for control group (*p* < 0.0001) and 1.754 mm^2^ for FHE group (*p* < 0.0001) (Figure 6B). Analysis of BV/TV (%) revealed a similar influence of BP. At 4 weeks, FHE + BP group’s percentage was 12.9% higher than the control group (FHE + BP = 42.77%, CON = 29.87%, *p* = 0.0002) and 8.986% higher than FHE group (FHE = 33.78%, *p* = 0.0129). Larger differences were recorded after 8 weeks with 46.15% for FHE + BP group, 30.83% for control group (*p* < 0.0001) and 32.93% for FHE group (*p* = 0.0001) being recorded (Figure 6C).

#### 3.3.4 Histological analysis and immunohistochemical staining

**FIGURE 7:**
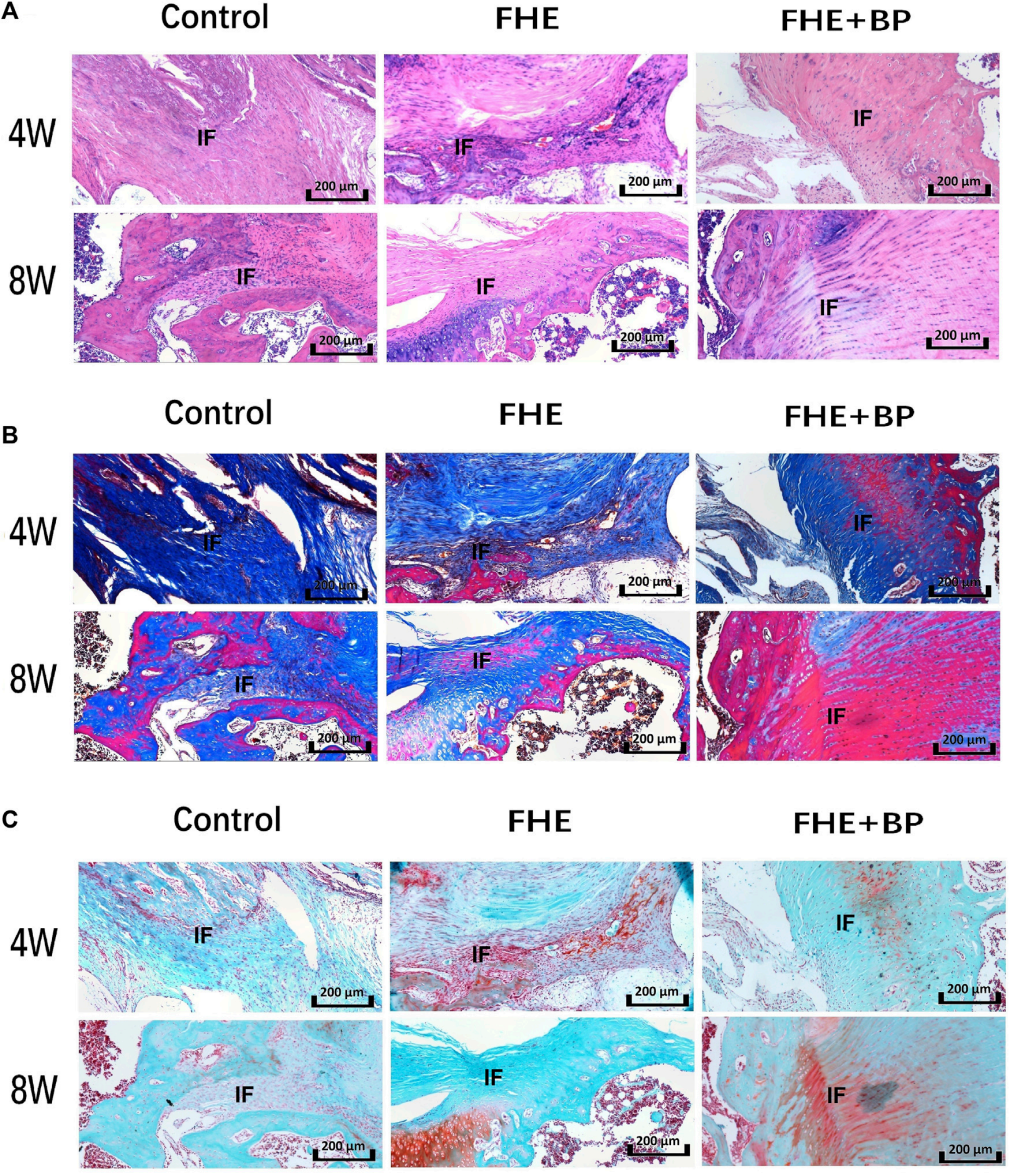
Results of histological analysis **(A)** Hematoxylin and eosin (H&E) staining **(B)** Masson’s trichrome staining and **(C)** Safranin O/fast green staining of the three groups at 4 and 8 weeks. IF: interface.

Additional histological analysis was further carried out to evaluate graft-bone interface (H&E staining (Figure 7A), Masson’s trichrome staining (Figure 7B) and Safranin O/fast green staining (Figure 7C). All three staining methods revealed better integration between the graft and bone tissues, judging by the cell morphology and distribution. HE staining showed an enhanced formation of graft within bone tissues in FHE + BP group while a new growth of bone tissues into fibrous tissues of graft was observed in both Masson’s trichrome staining (represented by purple color) and Safranin O/fast green staining (represented by red color).

**FIGURE 8:**
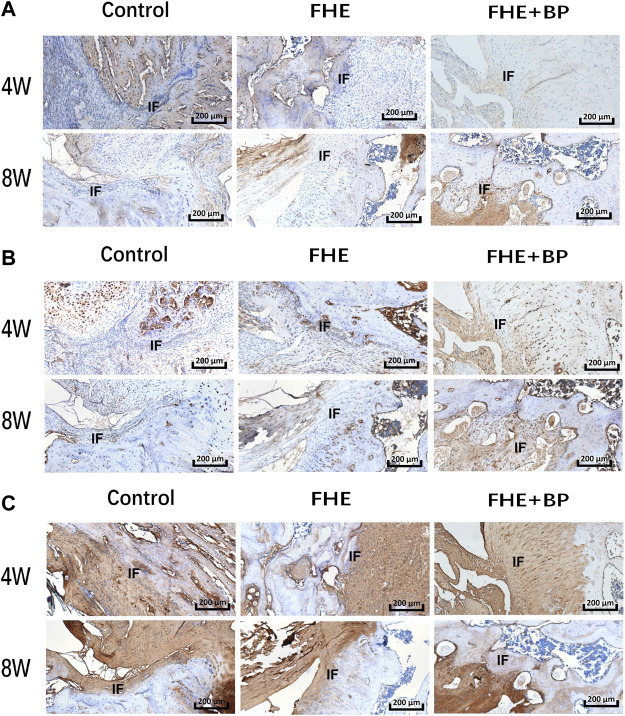
Results of immunohistochemical (IHC) analysis; IHC staining of **(A)** COL-I **(B)** BMP-2 and **(C)** COL-III in the three groups at 4 and 8 weeks. IF: interface.

Our *in vivo* investigation was concluded by IHC analysis of COL I (Figure 8A), BMP-2 (Figure 8B) and COL III (Figure 8C). Our results showed an increase in the expression of all targets in FHE + BP group after 8 weeks compared to CON and FHE groups. The enhanced integration and graft formation into bone tissues in FHE + BP group were represented by the higher positive staining (darker shades of brown) within and around the bone tissues, especially in COL I and COL III results. Both graft growth within bone tissues and bone growth within graft tissues can be observed.

**FIGURE 9:**
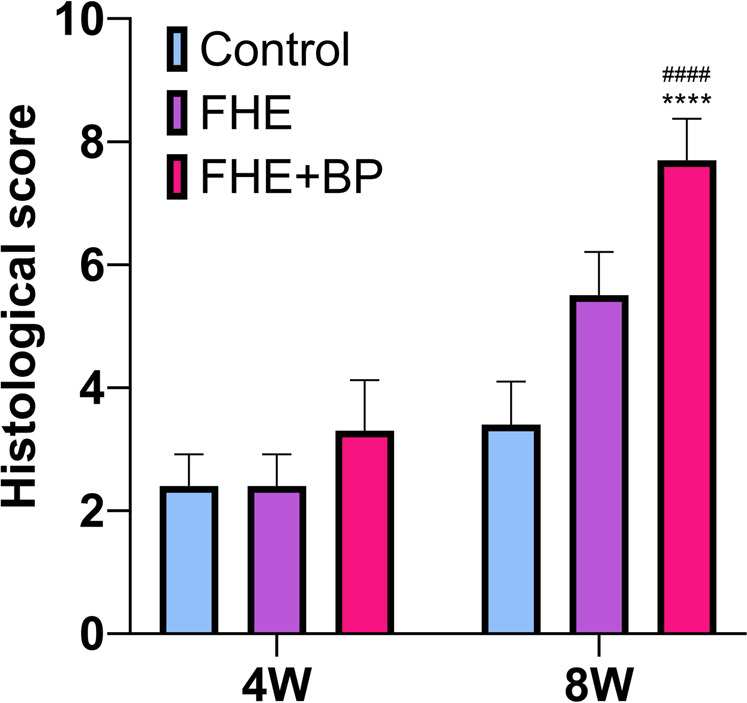
Histological score ****P < 0.0001 compare to control group, ^####^ *P* < 0.0001 compare to FHE group.

Overall, our analysis also revealed no significant difference at week 4 between groups’ average scores (CON: 2.4, FHE: 2.4, FHE + BP: 3.2). However, results at week 8 showed a significant improvement in the FHE + BP group (7.7 compared to FHE (5.5 *p* < 0.0001) and CON (3.4, *p* < 0.0001) groups (Figure 9).

## 4 Discussion

Hyaluronic acid (HA) is a biodegradable biomaterial with good biocompatibility and a hydrophilicity that plays an important role in cell adsorption, growth and differentiation. This biomaterial can be used as a temporary skeleton supporter and stimulator of new bone tissue growth. After a certain period of mechanical support, HA is gradually degraded and replaced by new bone tissue. However, the rapid biodegradation rate of HA does not match the tissue growth cycle (Khunmanee et al., 2017; Cai et al., 2023); thus, certain chemical modification is necessary.

The tendon bone interface repair in ligament section is a challenge in bone tissue engineering, since a loose interface between soft and hard tissues may result in inflammation. Therefore, the repair of the tendon bone interface requires injectable gels with good mechanical adaptation and regulation of the inflammatory microenvironment. Considering its unique hydromechanical properties, viscosity, water retention ability, physical properties, good biocompatibility, high viscoelasticity, permeability and plasticity, HA is a strong and common candidate in the field of tendon bone interface repair.

Considering the properties and advantages of HA, we designed and prepared a HA-based hydrogel to match the regenerative environment and effectively repair the tendon bone interface through Schiff base reaction combined with homogenized hybrid technology. First, HA was oxidized by NaIO_4_ into OHA. Since hypo-oxidation leads to poor gelatinization performance of prepared hydrogels while over-oxidation produces excessive toxic aldehyde group, the degree of oxidation had to be controlled artificially. Thus, NaIO_4_/HA ration and time of oxidation were strictly controlled. The successful synthesis of OHA was further proven by the analysis of HA absorption peak before and after modification through ^1^H-NMR spectrum test.

The various benefits and favorable characteristics of BP in tissues’ bioengineering made it a target of many relevant researches. In addition to clarifying BP’s relevant mechanisms and specific aspects, efforts have focused on identifying the best delivery vessel such as hydrogels (Huang et al., 2019; Miao et al., 2019; Xu et al., 2022b). However, no research has investigated the potential benefits of BP in ACLR recovery. Therefore, we aimed in this study to explore and illustrate the benefits of BP-FHE hydrogels in ACLR in both *in vitro* and *in vivo* settings.

First, BP nanoplates were homogenized with OHA and the presence of BP in OHA/BP composites hydrogels was confirmed through infrared spectrum test and FTIR spectrum. OHA molecule contains dialdehyde group, which can react with the primary amino group on the molecular chain of ε-polylysine (EPL) to form a reversible imine bond. The pore size analysis of the hydrogel with BP nanoflakes and the FHE hydrogel revealed no significant difference, indicating that BP is evenly dispersed in the FHE hydrogel. In short, FHE + BP Hydrogel has larger pore size, which is more conducive to cell growth and nutrient exchange. When applied in the tendon bone interface, it is more conducive to wound healing, and plays an effective role in filling and preventing potential loosening.

In addition, our findings showed an increase in water absorption of the hydrogel after adding BP, which may be due to the presence of BP which can form more mesh structures to store more water, thus improving the water absorption and swelling capacity of the hydrogel. Due to the inevitable loss of water (caused by the extension of time), the water locking capacity of the hydrogel with BP only increased slightly. It is speculated that the reason is that the groups on the molecules form hydrogen bond with the water molecules, increasing the intermolecular force and making the water molecules harder to volatilize, but since mixed BP made only 5%, no obvious difference in the water holding capacity was observed. It is very important to have good water absorption capacity for wound repair.

Our *in vitro* investigation used rBMSCs (Li et al., 2013). Cells were only used in further experiments after three propagations to ensure their quality and stability. First, the potential cytotoxicity of BP was investigated by CCK8 to analyze the biocompatibility and toxic effects of FHE + BP at different time points (1, 3 and 5 days) and compare it to similar features in FHE and control groups. Our results showed that compared to control group, both FHE and FHE + BP groups had significantly higher OD values at both day 3 and 5, while no significant differences were recorded at day 1. Thus, it is safe to assume that neither FHE nor BP result in any increased cytotoxicity and can be safely used in further experiments. The limited cytotoxicity of BP was further indicated in the live/dead staining analysis which showed stronger cell proliferation in FHE + BP group in comparison with the other two groups. The limited cytotoxicity not only introduces BP as a potential key element in bone healing and regeneration but also allows a comparison with other types of phosphorous which are usually associated with high toxicity that prevents any beneficial application in bioengineering (Gui et al., 2018).

Afterwards, our investigation focused on exploring the effect of BP on osteogenic differentiation through the ability to stimulate bone-like inorganic calcium deposition (ARS staining) and mRNA expression of COL1, OCN and RUNX-2 (RT-qPCR). The choice of these genes was based on their relevance to osteogenic activities (Miao et al., 2019; Cai et al., 2021b). Results of ARS staining analysis showed that FHE + BP group had significantly higher OD value when compared to FHE and control group, which indicates that BP can indeed stimulate and increase osteogenic differentiation and thus enhances bone-like inorganic calcium deposition. In addition, FHE + BP group was associated with higher levels of mRNA expression of all three included genes. Such results confirmed the potential role of BP in vitro settings on both RNA and cellular levels and indicated the need for further investigation in ACLR animal models.

Similar results have been reported by previous researches. For example, Li et al. (Li et al., 2021) observed a significant increase in mRNA expression of osteogenesis relevant genes after using BP based nanoparticles. Such influence has been associated by Bestami et al. (Bastami et al., 2017) with the ability of BP hydrogel to provide additional P5+ and therefore promote certain pathways like BMP-RUNX2. BP hydrogels’ influence on osteogenesis has also been attributed by Huang et al. (Huang et al., 2019) to their ability to enhance the release of phosphorus ions and capturing of calcium ions.

Since previous BP-relevant literature mainly focused on exploring the influence of BP on bone growth, most animal models were established through inflicting bone defects (Cheng et al., 2020). However, since our study focused on bone-tendon healing, our animal model was established through ACLR application. Starting by a macroscopic observation, we found no signs of infection in any of the animals in both groups which further confirmed the high standards and preventive measures carried through the procedure. Although no statistical significance was observed, macroscopic scores of FHE + BP group were higher than the other groups at both 4 weeks and 8 weeks. In addition, a larger difference was observed at 8 weeks, indicating BP’s extended positive effect. Such a positive influence of BP was further observed in our biomechanical testing which focused on failure load and stiffness of joints and revealed a significant difference in both parameters that favored FHE + BP group at both 4 and 8 weeks. Therefore, BP influence not only enhances the healing process but also strengthen the function recovery after reconstruction. Such findings are extremely important since they indicate the positive influence of BP application on biomechanical performance in weight-bearing sites, considering that the majority of researches focusing on bone-defect recovery usually choose bones in non-weight-bearing sites to simplify the process of recovery and minimize the complications (Miao et al., 2019).

To further evaluate the ability of BP to stimulate bone formation, micro-CT scanning of the tunnel was carried to analyze average bone tunnel area (mm^2^) and BV/TV (%). Our results showed that both parameters started to improve at week 4 but peaked at week 8. Such results not only indicate the BP effects but also illustrate the usefulness of FHE in guaranteeing a sustained release and effects for longer periods of times.

Further confirmation was provided by results of histological staining which showed an enhanced integration, bone and fibrocartilage formation in FHE + BP group. The different staining methods allowed a clear observation of cell morphology and distribution. Through HE staining, a new formation of graft cells can be observed in bone tissues, suggesting an enhanced integration in FHE + BP group, while the larger distribution of purple in Masson’s trichrome staining and red in Safranin O/fast green staining within graft tissues represent the formation of bone cells into graft. Such results clearly show that the positive influence of FHE + BP hydrogel is not limited to stimulating osteogenesis but also enhancing the tendon-bone healing.

Finally, IHC results showed increased expression of COL I, COL III and BMP-2. Again, higher positive staining (especially of COL I and COL III) in FHE + BP group represented by darker brown shades and clearer distribution within and around bone tissues indicates the graft growth and integration into the bone. The importance of COL I, COL III and BMP-2 has been investigated by other studies. Li et al. (Li et al., 2020) who investigated the application of tissue-engineering decellularized allografts for ACLR observed an increase in COL I and COL III expression followed by a decrease in COL III at month 3. As for BMP-2, an increase in expression was observed by Huang et al. (Huang et al., 2019) who investigated BP hydrogel scaffolds and their influence on bone generation rabbit animal models with bone defects.

Certain limitations partially affected the results of this study and can be addressed in future investigations. Our results can benefit from further investigations with larger sample-sizes and different animal models that can better mimic the microenvironment of human patients. Further analyses can also include wider range of osteogenesis-related markers to better represent all aspects of healing process and osteogenesis, such as ALP staining and protein electrophoresis data of phase osteogenesis. Non-etheless, our study is the first to show the beneficial role of BP in ACLR recovery and the usefulness of thermosensitive FHE hydrogel in guaranteeing long-term effects.

To conclude, BP-FHE hydrogels can successfully optimize the recovery of ACLR through enhancing osteogenesis and improving the integration of graft-bone interface. Such application provides histological and biomechanical benefits in addition to stimulating osteogenic differentiation on cellular levels.

## Data Availability

The original contributions presented in the study are included in the article/Supplementary Material, further inquiries can be directed to the corresponding author.
