# Supplementary material for: Injectable FHE+BP composites hydrogel with enhanced regenerative capacity of tendon-bone interface for anterior cruciate ligament reconstruction
Source: Front Bioeng Biotechnol. 2023 Feb 23;11:1117090. doi: 10.3389/fbioe.2023.1117090 (PMC9996450; doi:10.3389/fbioe.2023.1117090)
Supplement: Supplementary file 1 [file Table1.DOCX]

**Table S1** Macroscopic evaluation of ACL reconstruction

| Characteristic | Points |
| --- | --- |
| Integration at the insertion site with surrounding tissue |  |
| Complete | 2 |
| Minor disruption (<50% of area) | 1 |
| Major disruption (>50% of area) | 0 |
| Stiffness of the tendon graft |  |
| Normal compared to posterior cruciate ligament | 2 |
| Softer | 1 |
| Very soft | 0 |
| Appearance of the articular surface |  |
| Smooth | 2 |
| Fine fronds | 1 |
| Severe fronds | 0 |
| Color of the articular surface |  |
| Pearly, hyaline-like | 2 |
| Gloomy white | 1 |
| Yellow bone | 0 |
| Perfect score | 9 |

**Table S2** Scoring System for Histological Results

| Characteristic | Points |
| --- | --- |
| Fibrocartilage formation |  |
| Abundant | 3 |
| Moderate | 2 |
| Slight | 1 |
| None | 0 |
| New bone formation |  |
| Abundant | 3 |
| Moderate | 2 |
| Slight | 1 |
| None | 0 |
| Graft bonding to adjacent tissues |  |
| 75~100% | 3 |
| 50~75% | 2 |
| 25~50% | 1 |
| 0~25% | 0 |
| Perfect score | 9 |
